# The ROS/CaMK II/β-Catenin Signaling Axis Affects the Osteogenic Potential of BMSCs and Disrupts Implant Osseointegration: An In Vitro Study

**DOI:** 10.1155/ijod/5566776

**Published:** 2025-08-15

**Authors:** Jiaqi Ding, Kai Dong, Delong Niu, Wenjie Qiu, Wenjuan Zhou, Zhonghao Liu

**Affiliations:** ^1^The Affiliated Yantai Stomatological Hospital, Binzhou Medical University, Yantai 264000, China; ^2^Yantai Engineering Research Center for Digital Technology of Stomatology, Yantai 264000, China; ^3^Characteristic Laboratories of Colleges and Universities in Shandong Province for Digital Stomatology, Yantai 264003, China

**Keywords:** high glucose, osteogenic, ROS, Wnt/β-catenin pathway, Wnt/CaMK II pathway

## Abstract

**Objectives:** High glucose (HG)–induced oxidative stress affects implant osseointegration through various pathways. Oxidative stress is widely recognized to suppress the Wnt/β-catenin signaling pathway, thereby impairing bone metabolism and homeostasis. However, there are few reports on whether excessive reactive oxygen species (ROS) influence osteogenic differentiation of stem cells via the noncanonical Wnt/calmodulin-dependent protein kinase II (CaMK II) pathway. An investigation of the mechanism by which ROS/CaMK II/β-catenin signaling axis influences the osteogenic differentiation of bone marrow mesenchymal stem cells (BMSCs) on titanium surfaces is being carried out in this investigation.

**Materials and Methods:** In this study, titanium plates were specially treated to simulate implant surfaces. An osteogenic medium containing HG was used to cultivate BMSCs on titanium surfaces. The effects of excessive ROS induced by HG on the osteogenic differentiation of BMSCs, as well as on the expression of β-catenin and CaMK II, were examined using methods such as alkaline phosphatase (ALP) activity assay, quantitative real-time PCR (qRT-PCR), and immunofluorescence staining. Additionally, the effects of the Wnt/β-catenin and Wnt/CaMK II pathways on the osteogenesis of BMSCs on the titanium surface were observed by separately adding activators or inhibitors of β-catenin and CaMK II.

**Results:** Excessive ROS induced by HG inhibited osteogenic differentiation. In a HG environment, β-catenin expression decreased, while CaMK II expression increased. Moreover, we observed that activation of the Wnt/β-catenin pathway promoted osteogenesis, whereas activation of the Wnt/CaMK II pathway inhibited it.

**Conclusions:** In summary, BMSC osteogenesis on titanium surfaces is suppressed by HG–induced oxidative stress via the ROS/CaMK II/β-catenin signaling axis, which may subsequently impair implant osseointegration.

## 1. Introduction

Diabetes mellitus (DM) is a chronic systemic disease that poses a serious threat to human health. In addition to causing complications in cardiovascular, neurological, and renal systems, over 90% of diabetic patients also experience oral health issues, significantly affecting their overall health and general well-being. DM leads to a range of metabolic disorders, resulting in excessive oxidative stress, as well as elevated levels of inflammatory factors and advanced glycation end-products (AGEs) [1], which exacerbate periodontal inflammation and accelerate alveolar bone resorption [2]. Continuous alveolar bone resorption may even result in tooth loss [3].

Dental implants have become an excellent option for the treatment of dentition defects and edentulous patients. However, DM leads to reduced bone density, impaired osteogenesis, and delayed bone healing, ultimately compromising osseointegration and resulting in implant failure [4]. These processes have been reported to be associated with oxidative stress caused by hyperglycemia [5]. Essentially, oxidative stress involves an increase in reactive oxygen species (ROS) within cells. As critical inflammatory mediators, ROS can interfere with osteogenic differentiation and bone tissue regeneration through various signaling pathways, such as the Wnt pathway and RANK pathway [6, 7]. Wnt proteins can activate distinct intracellular signaling cascades by interacting with specific cell surface receptors. The most extensively studied among these is the canonical Wnt signaling pathway, commonly referred to as the Wnt/β-catenin pathway, which is characterized by its reliance on β-catenin-mediated transcriptional regulation. In contrast, noncanonical Wnt signaling pathways function independently of β-catenin and play crucial roles in various cellular processes. Two well-characterized noncanonical pathways include the Wnt/planar cell polarity (PCP) pathway and the Wnt/Ca^2+^ signaling pathway [8]. Among these, the canonical Wnt/β-catenin pathway serves as a key regulator of osteoblast proliferation and differentiation [9]. β-catenin, a crucial component of the Wnt signaling pathway, binds to T cell factor/lymphoid enhancer factor (TCF/LEF) transcription factors to act as a downstream effector molecule, initiating osteogenesis [10]. Studies suggest that the inhibition of the Wnt/β-catenin signaling pathway is strongly associated with bone loss and impaired bone metabolism function [11]. The lack of β-catenin leads to impaired maturation of osteoblasts [12], thereby disrupting osseointegration of implants [13]. However, a high level of ROS often inhibits the Wnt/β-catenin pathway, affecting stem cell differentiation and osteoblast activity, leading to impaired bone regeneration [14], poor osseointegration, and even implant failure [15–17].

In addition, Ca^2+^/calmodulin-dependent protein kinase II (CaMK II), as a crucial signaling molecule of the noncanonical Wnt pathway, also plays an important role in bone metabolism. Transforming growth factor-β (TGF-β) can affect osteoblast function via the CaMK II-SMAD axis, leading to delayed osteogenic differentiation and reduced collagen synthesis [18]. CaMK II downregulates TGF-β signaling by directly phosphorylating Smad2 and Smad4, which in turn impacts osteoblast development and function [19]. Additionally, CaMK II is a crucial molecule in intracellular Ca^2+^ signaling and RANKL-induced osteoclastogenesis [20]. Studies have shown that ROS has certain effects on CaMK II. For instance, excessive ROS generated by DM can continuously activate CaMK II by oxidizing the regulatory domain—M280/281—containing a pair of methionines, leading to increased expression of CaMK II under hyperglycemic conditions [21]. The impact of excessive ROS generated under high glucose (HG) conditions on CaMK II and its subsequent effects on the osteogenic differentiation of stem cells remains insufficiently explored, with limited studies addressing this specific relationship.

The investigation employs mouse bone marrow mesenchymal stem cells (BMSCs) as the research subject and simulates hyperglycemic conditions in vitro. The aim is to investigate the relationship between oxidative stress, CaMK II, and β-catenin in a HG environment and to explore the ROS/CaMK II/β-catenin signaling axis's impact on BMSCs' osteogenic potential. This research provides new theoretical insights into the pathogenesis of impaired osseointegration of implants in diabetic conditions, with potential clinical significance and application value.

## 2. Materials and Methods

### 2.1. Titanium Plates Preparation and Surface Topography Characterization

Pure titanium plates (diameter, 10 mm; thickness, 1 mm) were treated using turntable-type sandblasting equipment with 100-grit emery sand (Al_2_O_3_) media for sandblasting. After sandblasting, the titanium plates were cleaned with distilled water by ultrasonic washing, placed into a mixture of concentrated hydrochloric acid and concentrated sulfuric acid with a volume ratio of 2:1 and processed at a temperature of 65–75°C for 30 min. The titanium plates underwent ultrasonic cleaning with distilled water to ensure surface cleanliness [22]. Subsequently, we utilized a scanning electron microscope (Zeiss, Germany) to observe the surface morphology of all titanium plates after they were treated. The surface roughness of the titanium plates was measured using an optical interferometric 3D surface profiler. Prior to experimentation, we disinfected all plates using 75 wt% ethanol and ultraviolet (UV) irradiation.

### 2.2. Experimental Design

To determine whether DM regulates the conduction of the Wnt/CaMK II and Wnt/β-catenin pathways through oxidative stress, thus, affecting the osteogenesis of BMSCs on titanium surfaces, and to explore the related mechanism, the following three experiments were designed for the in vitro study.

The first one consisted of the normal glucose (NG) group, the HG group, the ROS-inhibited group, and the ROS-activated group: (a) NG: Osteogenic induction medium (OIM; Meilun Bio, Dalian, China) with NG concentration (5.5 mM D-glucose); (b) HG: OIM with HG concentration (35 mM D-glucose); (c) ROS inhibition: OIM (35 mM D-glucose) + Mito TEMPOL (20 μM; MCE, USA); (d) ROS activation: OIM (5.5 mM D-glucose) + H_2_O_2_ (25 μM; Lierkang, Shandong, China).

The second experiment included the NG group, the HG group, the CaMK II inhibition group, and the CaMK II activation group: (a) NG: OIM (5.5 mM D-glucose); (b) HG: OIM (35 mM D-glucose); (c) CaMK II inhibition: OIM (35 mM D-glucose) + KN-93 (10 μM; MCE, USA); (d) CaMK II activation: OIM (35 mM D-glucose) + Mito TEMPOL (20 μM) + CaCl_2_ (10 μM; MCE, USA). To isolate the specific effects of CaMK II activation within the noncanonical Wnt signaling pathway and eliminate confounding factors, the ROS inhibitor Mito TEMPOL was added to the CaMK II-activated group. This approach was employed to neutralize the deleterious influence of oxidative stress on osteogenesis, thereby ensuring that the observed experimental outcomes were attributable solely to the activation of CaMK II signaling and not to oxidative stress-induced artifacts.

The third experiment included the NG group, the HG group, the β-catenin activation group, and the β-catenin inhibition group: (a) NG: OIM (5.5 mM D-glucose); (b) HG: OIM (35 mM D-glucose); (c) β-catenin activation: OIM (35 mM D-glucose) + Wnt-3a (100 ng/mL; Abcam, Britain); (d) β-catenin inhibition: OIM (35 mM D-glucose) + Mito TEMPOL (20 μM) + DKK1 (0.2 μg/mL; YESEN, Shanghai, China). To ensure experimental specificity and eliminate confounding effects, the ROS inhibitor Mito TEMPOL was added to the β-catenin-inhibited group. This step was taken to suppress oxidative stress-related interference with osteogenesis, thereby confirming that the observed effects were exclusively attributable to the inhibition of β-catenin signaling within the canonical Wnt signaling pathway.

### 2.3. Cell Culture

BMSCs from C57BL/6 mice were obtained from Meilun Bio (Dalian, China). At 1250 rpm for 4 min, the BMSCs stored at −80°C were subjected to centrifugation. The supernatant was carefully decanted. BMSCs were carefully resuspended in fresh complete culture medium, which consisted of Dulbecco's modified eagle medium (DMEM; Meilun Bio, Dalian, China) supplemented with 10% fetal bovine serum (FBS; Meilun Bio, Dalian, China) and 1% penicillin–streptomycin (Meilun Bio, Dalian, China). A single-cell suspension was prepared and seeded in T25 cell culture flasks, followed by incubation at 37°C in 5% CO_2_. Nonadherent cells were washed out with phosphate-buffered saline (PBS; Meilun Bio, Dalian, China) each 3 days. Adherent cells were cultured until they reached 80% confluency and then passaged. Cells of Passages 3–5 were used for subsequent experiments.

### 2.4. Analysis of Cell Morphology on the Titanium Plates

BMSCs were seeded onto titanium plates. After 24 h, DAPI (Meilun Bio, Dalian, China)/phalloidin (KeyGEN BioTECH, Jiangsu, China) fluorescence double staining was used to observe cell morphology. The fixed monolayer cells were incubated with phalloidin-conjugated fluorescent probes (100 µM) for 20 min to label the cytoskeleton. Next, the phalloidin working solution was removed and the DAPI working solution (1 µg/mL) was added to the cells for 5 min to label the nucleus. Finally, after rinsing, stained cells were visualized using confocal microscopy (Leica, Germany).

### 2.5. Cell Counting Kit-8 (CCK-8)

We seeded 5000 BMSCs per well in 96-well plates. After 12 h of cell adhesion, HG medium containing 0, 5, 10, 20, 30, or 40 μM Mito TEMPOL or 0, 5, 10, 20, or 40 μM KN-93 was added to the wells, respectively. Three replicate wells were set for each concentration. Cell viability was measured using CCK-8 (Beyotime, Shanghai, China) assays after culture for 24, 48, and 72 h. For this, each well of plates received 10 μL CCK-8 solution, followed by incubation of the plates at 37°C for 2 additional hours. After incubation, we used a plate reader (Thermo, USA) at 450 nm to measure optical density (OD) value. An evaluation of experimental drugs' impacts on cell proliferation was performed based on the collected data.

### 2.6. Live/Dead Cell Staining

Cells were seeded into six-well plates at a density of 5 × 10^4^ cells/mL. After 24 h of incubation, HG medium supplemented with 0, 5, 10, or 20 μM Mito TEMPOL was added to the wells, respectively. Three replicate wells were included for each concentration group. After 48 h of treatment, a live/dead cell staining kit was used to evaluate the effect of Mito TEMPOL on BMSC viability. Following PBS washing, a staining solution containing propidium iodide (PI) and Calcein-AM was added to each well. The cells were incubated in the dark for 25 min, and then, observed under a fluorescence microscope to distinguish between live and dead cells.

### 2.7. ROS Assay

As part of the ROS detection kit used in this experiment, dichloro-dihydro-fluorescein diacetate (DCFH-DA; Meilun Bio, Dalian, China) acts as a fluorescent marker for detecting ROS in cells. First, cells were seeded on confocal dishes and cultured until 85% confluence was reached. HG media containing 0, 5, 10, 20, and 40 μM Mito TEMPOL were added, respectively. Three replicate wells were set for each concentration. After culturing for 24 h, the original medium was carefully aspirated. A total of 2 mL DMEM without serum supplemented with 10 μM DCFH-DA (Meilun Bio, Dalian, China) was added to the BMSCs. This was followed by a 30-min dark incubation at 37°C. Subsequently, confocal microscopy (Leica, Germany) was employed to observe the fluorescence intensity of BMSCs after three washes with PBS. Images were captured and ImageJ was used to quantify fluorescence intensity.

After determining the appropriate concentration of Mito TEMPOL, BMSCs were seeded onto titanium plates placed in confocal culture dishes. After being cultured for 7 days under the conditions assigned to each group in the first part of the experiment. We removed the medium and added 2 mL DMEM without serum supplemented with 10 µM DCFH-DA. This was followed by a 30-min dark incubation at 37°C. Subsequently, confocal microscopy (Leica, Germany) was employed to observe the fluorescence intensity of BMSCs after three washes with PBS. Fluorescent cells were manually counted from the images using ImageJ and fluorescence intensity was quantified for each group.

### 2.8. Alkaline Phosphatase (ALP) Activity Assay

BMSCs were seeded onto titanium plates, and osteogenic induction was performed according to the culture conditions of each experimental group. Three replicate wells were set for each group. Days 3, 7, and 14 were chosen for the ALP activity assay. The confluent monolayer of cells was lysed on ice using western and IP cell lysis buffer (Beyotime, Shanghai, China), and the lysates were gathered and subjected to centrifugation at 12,000 rpm for 5 min. ALP activity in the cell lysates was measured with an ALP assay kit (Beyotime, Shanghai, China). The ALP activity in the samples was determined based on standard definition of enzyme activity.

### 2.9. Quantitative Real-Time PCR (qRT-PCR)

BMSCs were seeded onto titanium plates and cultured under osteogenic conditions for 10 days according to the specific protocols of each experimental group to assess the levels of osteogenic gene expression. Three replicate wells were set for each group. TRIzol (KeyGEN BioTECH, Jiangsu, China) was used to extract the total RNA. Complementary deoxyribonucleic acid (cDNA) was synthesized using a cDNA synthesis kit (Cwbio, China) to detect the expression of osteopontin (OPN), type I collagen (COL-1), runt-related transcription factor 2 (Runx2), β-catenin, and CaMK II. Glyceraldehyde-3-phosphate dehydrogenase (GAPDH) served as the reference. Primer sequences for qRT-PCR are listed in Table 1.

### 2.10. Immunofluorescence Staining

OPN, COL-1, Runx2, β-catenin, and CaMK II protein expression were detected through immunofluorescence staining. BMSCs at Passage 3 were seeded on a titanium plate. Osteogenic induction was performed for 10 days according to the culture conditions of each experimental group, with three replicate wells set for each group. Subsequently, a 4% (*w*/*v*) paraformaldehyde solution prepared in PBS (Macklin, Shanghai, China) was used to fix the BMSCs at room temperature for 30 min. The titanium plates were washed with PBS, followed by incubation in 0.5% Triton X-100 (Beyotime, Shanghai, China) at 37°C for 20 min. Subsequently, they were rinsed three times with PBS and blocked using goat serum (Solarbio, Beijing, China). An overnight incubation was performed at 4°C with the antibodies of OPN, COL-1, Runx2, β-catenin, and CaMK II proteins, respectively. Afterwards, cells were washed in PBS and exposed to the IgG antibody (Beyotime, Shanghai, China) solution at 37°C for 60 min. Incubation was conducted at room temperature in the dark for 30 min with FITC-labeled phalloidin (ABclonal, Wuhan, China) after three PBS washes. We then washed the cells twice with PBS, incubated them with DAPI for 5 min, and then, viewed the fluorescent micrographs under a microscope (Leica, Germany).

### 2.11. Statistical Analysis

All experiments were repeated three times to ensure accuracy. The experimental results were presented as mean ± standard deviation (SD). Significant differences were compared using the one-way ANOVA with multiple comparison tests or Student's *t*-test and performed with GraphPad Prism 10. *p*  < 0.05 denotes a statistically significant difference.

## 3. Results

### 3.1. Topographies and Surface Cell Morphology of Titanium Plates

**Figure 1:**
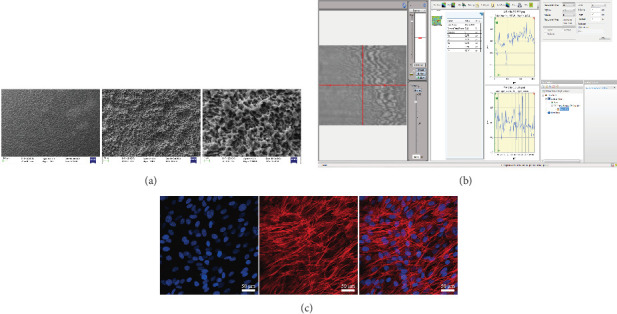
Titanium plates can be good carriers for bone marrow mesenchymal stem cells (BMSCs). (a) Microscopic surface of titanium plates, (b) surface roughness of titanium plates, and (c) cytoskeleton and nuclear staining.

At a magnification of 2000x under SEM, the surface of the treated titanium plates exhibited an irregular rough texture, with a surface roughness measured at 0.173 μm (Figure 1a). This surface structure effectively simulated the morphology of implant surfaces, providing a favorable microenvironment for cell adhesion and growth (Figure 1b). Titanium surfaces treated with acid etching and sandblasting showed excellent cell adhesion properties, with dense cell distribution in clusters and colonies. The cells exhibited a healthy morphology, extending pseudopodia to adhere to the titanium surfaces. This demonstrated that the treated micro-rough titanium surfaces could serve as an effective cell carrier.

### 3.2. Evaluation of Cytotoxic and Proliferative Effects of Mito TEMPOL on BMSCs

**Figure 2:**
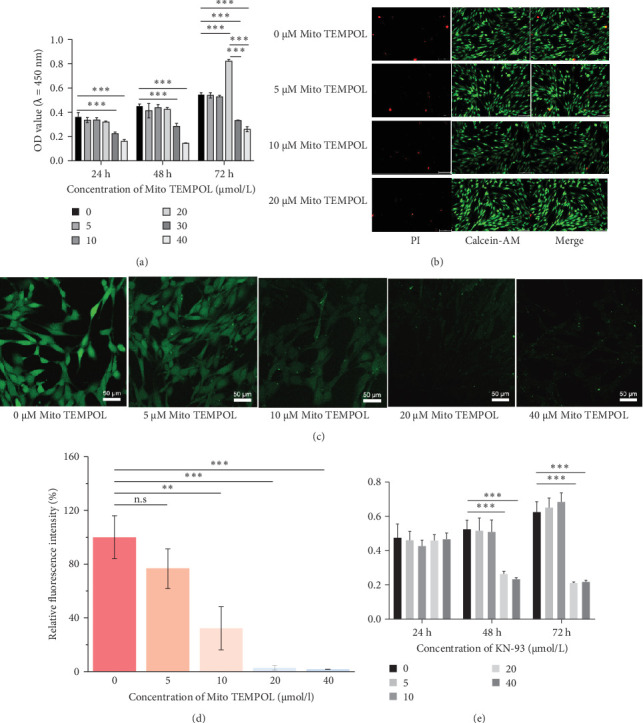
Determination of optimum concentration of KN-93 and Mito TEMPOL. (a) The Cell Counting Kit-8 (CCK-8) assay revealed that a concentration of 30 and 40 μM Mito TEMPOL impaired the proliferation capacity of BMSCs. *⁣*^*∗*^*p* < 0.05; *⁣*^*∗∗*^*p* < 0.01; *⁣*^*∗∗∗*^*p* < 0.001. (b) Live/dead cell staining demonstrated that Mito TEMPOL at concentrations ≤20 μM did not exhibit significant cytotoxicity toward BMSCs. (c) The ROS assay determined that the optimal concentration of Mito TEMPOL for inhibiting ROS was 20 μM. (d) Quantitative analysis of fluorescence intensity determined that the optimal concentration of Mito TEMPOL for ROS inhibition was 20 μM. ⁣^*∗*^*p* < 0.05; *⁣*^*∗∗*^*p* < 0.01; *⁣*^*∗∗∗*^*p* < 0.001. (e) The CCK-8 assay revealed that 20 and 40 μM concentrations of KN-93 impaired the proliferation capacity of BMSCs at 48 and 72 h. *⁣*^*∗*^*p* < 0.05; *⁣*^*∗∗*^*p* < 0.01; *⁣*^*∗∗∗*^*p* < 0.001.

To investigate the cytotoxic effects of Mito TEMPOL on BMSCs, cells were treated with a HG medium containing varying concentrations of Mito TEMPOL for 1, 2, and 3 days. CCK-8 was then used to measure cell viability. According to the results, except for 30 and 40 µM Mito TEMPOL, which affected cell proliferation, other concentrations of Mito TEMPOL showed no inhibitory effect on cell viability compared to the control group without Mito TEMPOL (Figure 2a).

Live/dead cell staining was performed to further evaluate whether Mito TEMPOL exerts cytotoxic or other adverse effects on BMSCs. Live cells with esterase activity exhibited green fluorescence, whereas dead cells with compromised plasma membranes showed red fluorescence. Minimal red fluorescence was observed across all groups, indicating a very low number of dead cells and confirming that Mito TEMPOL did not induce significant cytotoxicity. Furthermore, the number of green fluorescent cells in the ≤20 μM Mito TEMPOL groups showed no significant difference compared to the control group and the cells maintained normal morphology. These observations are consistent with the results of the CCK-8 proliferation assay and further support that Mito TEMPOL at concentrations ≤20 μM does not exert appreciable cytotoxicity on BMSCs.

### 3.3. The Optimal Concentration of Mito TEMPOL for ROS Inhibition Was 20 μM

Due to the limited existing research on the application of Mito TEMPOL in C57 murine BMSCs, this study employed a ROS assay to determine the optimal concentration of Mito TEMPOL for inhibiting excessive ROS generated in BMSCs in a HG environment. It was observed that the lowest ROS fluorescence intensity occurred at Mito TEMPOL concentrations of 20 and 40 μM (Figure 2c,d). Consequently, 20 μM Mito TEMPOL was selected for this experiment.

### 3.4. Evaluation of Cytotoxic and Proliferative Effects of KN-93 on BMSCs

The cytotoxicity and proliferative effects of KN-93 on BMSCs were assessed using a CCK-8 assay. We incubated the BMSCs in a HG medium containing varying concentrations of KN-93. After 24 h of incubation, no significant difference was observed in BMSC viability across all KN-93 concentrations. However, after 48 h, KN-93 at concentrations of 20 and 40 µM significantly inhibited BMSCs viability. Therefore, a concentration of 10 µM KN-93 was used to inhibit CaMK II (Figure 2e).

### 3.5. BMSCs on the Titanium Surfaces Produced Excessive ROS in a HG Environment

**Figure 3:**
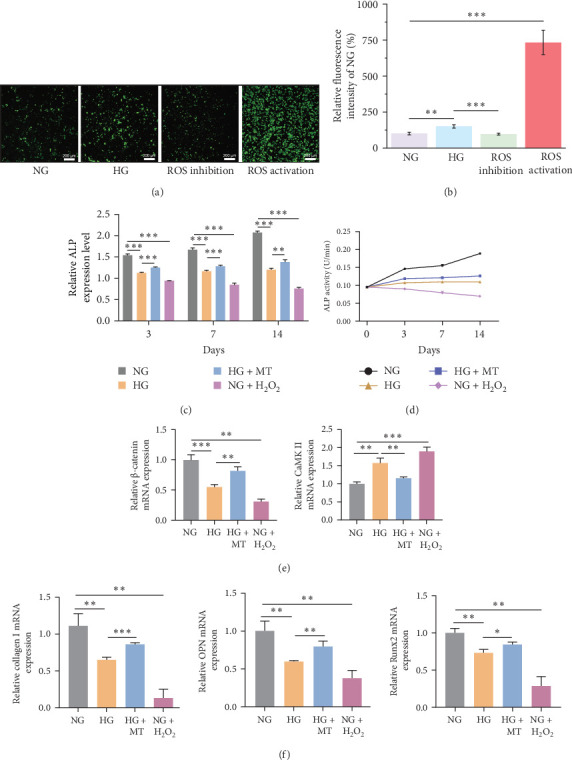
Oxidative stress, alkaline phosphatase (ALP) activity, and osteogenic gene assays for the first part of the experiment revealed that high glucose-induced ROS overproduction led to impaired osteogenic differentiation of titanium-surfaced BMSCs. (a) Oxidative stress analysis in the first part of the experiment revealed that a high-glucose environment induced excessive ROS generation in BMSCs cultured on titanium surfaces. (b) Quantitative analysis of fluorescence intensity for oxidative stress detection in the first part of the experiment. *⁣*^*∗*^*p* < 0.05; *⁣*^*∗∗*^*p* < 0.01; *⁣*^*∗∗∗*^*p* < 0.001. (c, d) ALP activity assays in the first part of the experiment revealed that excessive ROS production induced by high glucose resulted in reduced ALP activity in BMSCs cultured on titanium surfaces. *⁣*^*∗*^*p* < 0.05; *⁣*^*∗∗*^*p* < 0.01; *⁣*^*∗∗∗*^*p* < 0.001. (e) Quantitative real-time PCR (qRT-PCR) analysis of *β-catenin* and *CaMK II* genes revealed that excessive ROS production induced by high glucose activated the expression of the CaMK II signaling molecule while inhibiting the expression of the β-catenin signaling molecule. *⁣*^*∗*^*p* < 0.05; *⁣*^*∗∗*^*p* < 0.01; *⁣*^*∗∗∗*^*p* < 0.001. (f) qRT-PCR analysis of osteogenic genes demonstrated that excessive ROS production induced by high glucose resulted in decreased expression of osteogenic genes in BMSCs cultured on titanium surfaces. *⁣*^*∗*^*p* < 0.05; *⁣*^*∗∗*^*p* < 0.01; *⁣*^*∗∗∗*^*p* < 0.001.

ROS levels in BMSCs were measured via assessing fluorescence intensity, as oxidative stress is a key factor in DM. The HG group exhibited a 1.5-fold increase in DCF fluorescence intensity relative to the NG group (9.91 ± 12.08 vs. 6.62 ± 9.05; *p*  < 0.01). Compared to NG, ROS-activated group levels were 7.3-fold higher (48.53 ± 84.56 vs. 6.62 ± 9.05; *p*  < 0.001). The DCF fluorescence intensity in the HG group was 1.5 times higher than that observed in the ROS-inhibited group (9.91 ± 12.08 vs. 6.35 ± 6.27; *p*  < 0.001; Figure 3a,b). It appeared that HG levels led to excessive ROS production.

### 3.6. HG–Induced ROS Overproduction Led to Impaired Osteogenic Differentiation of Titanium-Surfaced BMSCs

**Figure 4:**
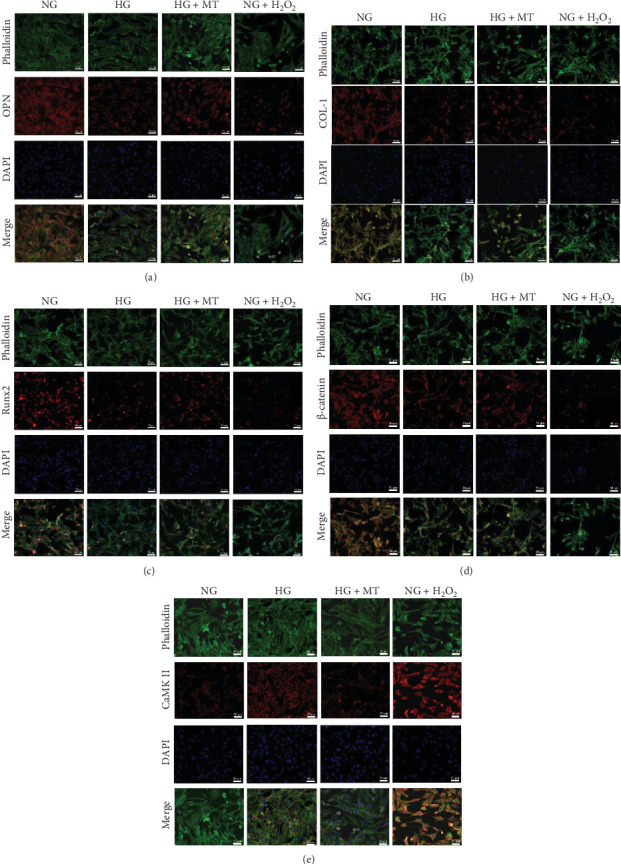
Immunofluorescence staining in the first part of the experiment demonstrated that excessive ROS production induced by high glucose resulted in decreased expression of osteogenic proteins, β-catenin, and CaMK II proteins in BMSCs cultured on titanium surfaces. (a) Immunofluorescence staining of osteopontin (OPN) protein in the first part of the experiment. (b) Immunofluorescence staining of type I collagen (COL-1) protein in the first part of the experiment. (c) Immunofluorescence staining of runt-related transcription factor 2 (Runx2) protein in the first part of the experiment. (d) Immunofluorescence staining of β-catenin protein in the first part of the experiment. (e) Immunofluorescence staining of CaMK Il protein in the first part of the experiment.

The osteogenic differentiation capacity of BMSCs was assessed through ALP activity assay, qRT-PCR, and immunofluorescence staining. On Days 3, 7, and 14 of osteogenic induction, the ALP level in the HG group was markedly reduced compared to the NG group, whereas the ROS inhibition group exhibited increased ALP activity relative to the HG group (Figure 3c,d). The ROS activation group exhibited the weakest ALP activity. Additionally, qRT-PCR and immunofluorescence staining indicated that, on Day 10, the mRNA and protein expression of COL-1, Runx2, and OPN were downregulated under HG conditions relative to NG conditions. The ROS activation group demonstrated the lowest expression of osteogenic genes and proteins. The mRNA and protein expression of Col-1, Runx2, and OPN were upregulated in the ROS inhibition group relative to the HG group (Figures 3f and 4a–c). These results indicated that excessive ROS induced by a HG environment inhibited the osteogenic differentiation of BMSCs.

### 3.7. HG–Induced ROS Overproduction Activated CaMK II Signaling and Inhibited β-Catenin Signaling

Since oxidative stress regulates cellular signaling through both canonical and noncanonical Wnt pathways, the expression of key proteins, β-catenin and CaMK II, in these pathways was investigated under HG conditions. In a HG environment, CaMK II mRNA and protein expression were upregulated in BMSCs on titanium surfaces, while β-catenin mRNA and protein expression decreased. This trend was consistent with the expression changes of β-catenin and CaMK II observed in BMSCs treated with the ROS activator, H_2_O_2_. Conversely, in BMSCs treated with the ROS inhibitor Mito TEMPOL, the β-catenin mRNA and protein levels were upregulated, while those of CaMK II were downregulated (Figures 3e and 4d,e). These findings suggested that a HG environment activated the expression of CaMK II signaling molecules and inhibited β-catenin signaling molecules in titanium surfaces BMSCs through excessive ROS.

### 3.8. Wnt/CaMK II Pathway Inhibited Osteogenic Differentiation of BMSCs on Titanium Surfaces

**Figure 5:**
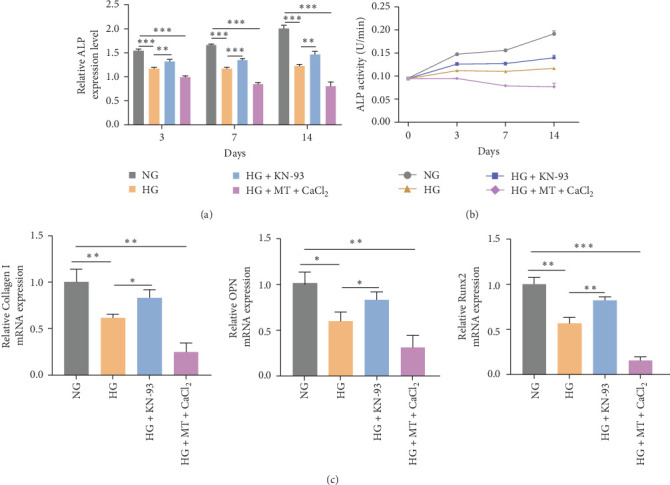
ALP activity and osteogenic gene assays for the second part of the experiment revealed that Wnt/CaMK II pathway inhibited osteogenic differentiation of BMSCs on titanium surfaces. (a, b) ALP activity assays in the second part of the experiment indicated that activation of the Wnt/CaMK II pathway led to a reduction in ALP activity in BMSCs cultured on titanium surfaces. *⁣*^*∗*^*p* < 0.05; *⁣*^*∗∗*^*p* < 0.01; *⁣*^*∗∗∗*^*p* < 0.001. (c) qRT-PCR of osteogenic genes in the second part of the experiment revealed that activation of the Wnt/CaMK II pathway resulted in decreased expression of osteogenic genes in BMSCs cultured on titanium surfaces. *⁣*^*∗*^*p* < 0.05; *⁣*^*∗∗*^*p* < 0.01; *⁣*^*∗∗∗*^*p* < 0.001.

**Figure 6:**
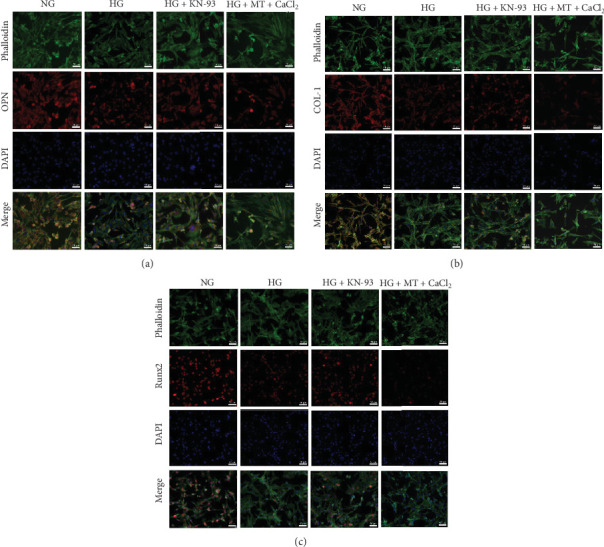
Immunofluorescence staining in the second part of the experiment revealed that activation of the Wnt/CaMK II pathway led to decreased expression of osteogenic proteins in BMSCs cultured on titanium surfaces. (a) Immunofluorescence staining of OPN protein in the second part of the experiment. (b) Immunofluorescence staining of COL-1 protein in the second part of the experiment. (c) Immunofluorescence staining of Runx 2 protein in the second part of the experiment.

The first part of the experiment has demonstrated that excessive ROS induced by HG stimulates CaMK II production. To gain a deeper understanding of how the Wnt/CaMK II pathway influenced osteoblast differentiation in BMSCs on titanium plates under HG conditions, the second part of the experiment was conducted. Results from the ALP activity assays on Days 3, 7, and 14 of osteogenic induction revealed that CaMK II inhibition group exhibited significantly higher ALP activity compared to HG group, whereas CaMK II activation group showed lower ALP activity compared to NG group (Figure 5a, b). After 10 days of osteogenic induction, the CaMK II inhibition group led to elevated mRNA and protein levels of osteogenic markers (Col-1, Runx2, and OPN) compared with HG group. Contrary to the NG group, CaMK II activation decreased the expression of osteogenic-related genes and proteins (Figures 5c and 6a–c). It suggested that the Wnt/CaMK II pathway was also involved in the regulation of the osteogenic differentiation of BMSCs, inhibiting their osteogenesis.

### 3.9. BMSC Osteogenesis on Titanium Surfaces Was Enhanced by the Wnt/β-Catenin Pathway

**Figure 7:**
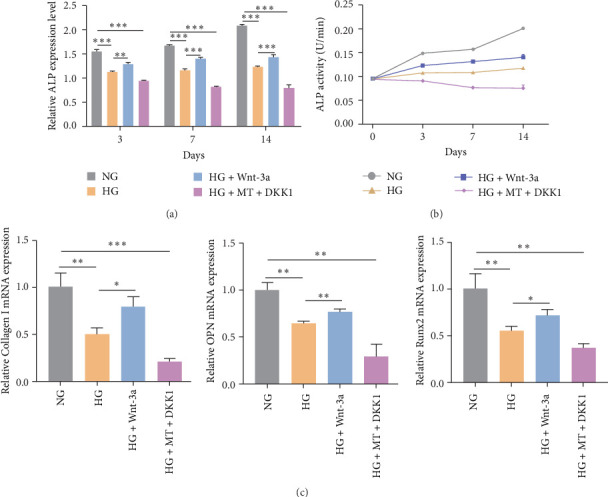
ALP activity and osteogenic gene assays in the third part of the experiment demonstrated that the Wnt/β-catenin pathway promoted the osteogenic differentiation of BMSCs on titanium surfaces. (a, b) ALP activity assays in the third part of the experiment demonstrated that activation of the Wnt/β-catenin pathway increased the ALP activity of BMSCs cultured on titanium surfaces. *⁣*^*∗*^*p* < 0.05; *⁣*^*∗∗*^*p* < 0.01; *⁣*^*∗∗∗*^*p* < 0.001. (c) qRT-PCR of osteogenic genes in the third part of the experiment revealed that activation of the Wnt/β-catenin pathway increased the expression of osteogenic genes in BMSCs cultured on titanium surfaces. *⁣*^*∗*^*p* < 0.05; *⁣*^*∗∗*^*p* < 0.01; *⁣*^*∗∗∗*^*p* < 0.001.

**Figure 8:**
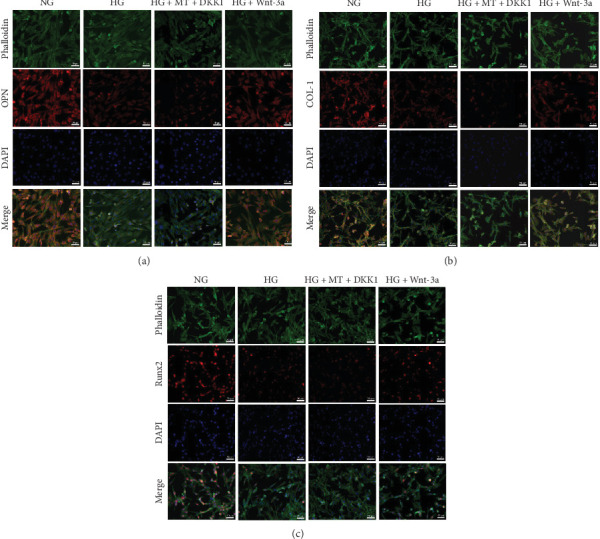
Immunofluorescence staining in the third part of the experiment revealed that activation of the Wnt/β-catenin pathway increased the expression of osteogenic proteins in BMSCs cultured on titanium surfaces. (a) Immunofluorescence staining of OPN protein in the third part of the experiment. (b) Immunofluorescence staining of COL-1 protein in the third part of the experiment. (c) Immunofluorescence staining of Runx 2 protein in the third part of the experiment.

In order to determine whether Wnt/β-catenin pathway affects osteogenic differentiation of BMSCs on titanium surfaces under HG conditions, ALP activity was assessed on Days 3, 7, and 14 following osteogenic induction. The results demonstrated an increase in ALP activity in β-catenin activation group in comparison to the HG group. Conversely, the β-catenin inhibition group exhibited reduced ALP activity relative to the NG group (Figure 7a,b). Additionally, qRT-PCR and immunofluorescence staining demonstrated that the mRNA and protein expressions of COL-1, Runx2, and OPN were elevated in the β-catenin activation group in comparison to the HG group, while the β-catenin inhibition group exhibited lower expression of these markers relative to the NG group (Figures 7c and 8a–c). These findings collectively suggested that the Wnt/β-catenin signaling pathway promoted the osteogenic differentiation of BMSCs on titanium surfaces.

## 4. Discussion

The HG environment in diabetic patients is a critical factor contributing to their dysfunction [23]. Hyperglycemia leads to the accumulation of AGEs, which bind to AGE receptors, producing excess ROS [24]. This cascade subsequently triggers the activation of downstream signaling pathways, inhibiting cell growth, mineralization, and the production of key markers of osteogenesis, including Runx2, COL-1, as well as osteocalcin (OC) [25], ultimately disrupting bone metabolism [26]. ALP activity marks osteogenic differentiation and serves as a critical marker of osteogenic activity. RUNX2, COL-1, and OPN play key roles in the early stages of osteogenesis. Therefore, this study measured ALP activity along with the expression of these osteogenic genes and their protein products as indicators of osteogenic differentiation. Aligned with prior research, this study demonstrated a reduction in ALP activity, accompanied by diminished expression of osteogenic genes and proteins, in a HG environment. The results strongly indicate that the osteogenic potential of BMSCs on titanium surfaces is significantly suppressed under such conditions. Regarding the selection of glucose concentration, most existing studies [27, 28] have employed culture media containing 5.5 and 25.5 mmol/L glucose to simulate normoglycemic and hyperglycemic conditions, respectively. However, our preliminary observations indicated that a moderate elevation in glucose concentration could enhance BMSC proliferation and paradoxically promote osteogenic differentiation. This finding is consistent with previous reports [29]. Therefore, to better mimic a pathological hyperglycemic environment that adversely affects osteogenesis, we adopted a higher glucose concentration of 35 mmol/L, as supported by earlier literature [30]. Under these conditions, we confirmed that the osteogenic differentiation of BMSCs was significantly inhibited, validating the suitability of this model for studying hyperglycemia-induced impairment in bone formation.

Bone formation is an important step of implant osseointegration. It is a complex process that involves the differentiation of BMSCs into preosteoblasts, followed by osteoblastic proliferation, differentiation, and mineralization. Oxidative stress caused by hyperglycemia significantly impairs osteoblast activity while disrupting the regeneration of bone tissue, resulting in poor osseointegration or potential implant instability [31]. ROS, the primary products of oxidative stress, are closely related to bone metabolism. While ROS at physiological levels act as specific signaling molecules regulating various pathways, excessive ROS notably inhibit the osteogenic differentiation of BMSCs [17], induce osteoblast damage, and ultimately lead to osteoblast apoptosis [32]. In eukaryotic cells, mitochondrial oxidative phosphorylation is the main source of ROS production [33]. Studies have shown that DM disrupts mitochondrial homeostasis, causing excessive ROS accumulation, which subsequently triggers abnormal bone metabolism [16]. Therefore, we believe that antioxidant therapy may offer a promising approach for treating DM-induced osseointegration deficiencies. The findings of this study are consistent with the views mentioned above. The results of this study showed that a HG environment significantly increased ROS production and had a negative impact on BMSC osteogenesis, while Mito TEMPOL partially reversed these effects.

ROS in BMSCs modulates osteogenic differentiation through the Wnt/β-catenin signaling pathway [34]. This study revealed a notable reduction in β-catenin expression in cells treated with a ROS activator relative to those under NG conditions. Conversely, cells treated with ROS inhibitors exhibited increased β-catenin expression compared to those in the HG condition. This indicates that excessive ROS induced by HG inhibits the Wnt/β-catenin signaling pathway, aligning with findings from most current studies [35–37]. As a result of ROS activating JNK, JNK phosphorylates transcription factors of TCF, decreasing β-catenin/TCF's ability to bind DNA. The Wnt/β-catenin signaling pathway is fundamental to regulating the osteogenic differentiation of stem cells. It is crucial for directing the conversion of BMSCs into osteoblasts, promoting the deposition of minerals in these osteoblasts, as well as facilitating the development of the bone matrix. As part of the Wnt/β-catenin signaling cascade, β-catenin, once stably expressed, translocates to the cell's core. There, β-catenin interacts with TCF/LEF transcription factors. As a result of this interaction, Runx2 is activated [38], a key factor that governs the early stages of the osteogenic differentiation process. Runx2 plays a pivotal role in driving the activation of genes specific to osteoblasts, including ALP, OC (BGP), as well as collagen type I (COL-1) [39], thus, promoting the maturation of osteoblasts. It is known that Wnt-3a activates the Wnt/β-catenin pathway. It binds to Frizzled family receptors and LRP5/6 coreceptors, becoming activated intracellularly and further inhibiting GSK-3β activity. This allows β-catenin to stabilize and translocate into the nucleus [40], where it activates the transcription of downstream osteogenic genes such as Runx2 and Osterix. Runx2 acts as a key transcriptional mediator of the canonical Wnt signaling pathway and is critically involved in the regulation of osteoblast differentiation and maturation, including the expression of osteogenic markers such as OPN, OC, COL-1, osteoprotegerin (OPG), et cetera [41]. Osterix, an osteoblast-specific transcription factor functioning downstream of Runx2, further contributes to osteogenic differentiation by regulating the expression of COL-1, OC, and ALP [42]. This was demonstrated in the present study, the addition of Wnt-3a in a HG environment significantly upregulated osteogenic genes and proteins and enhanced ALP activity in the β-catenin activation group compared to the HG group. Although Wnt-3a is known to promote osteogenic differentiation of stem cells via activation of the canonical Wnt/β-catenin signaling pathway, ROS can, to a certain extent, suppress Wnt-3a expression. This suppression subsequently leads to the activation of GSK-3β through phosphorylation, which promotes the degradation of β-catenin. As a result, the nuclear translocation of β-catenin is reduced, thereby downregulating the expression of key osteogenic transcription factors such as Runx2 and Osterix. Consequently, the expression of osteogenesis-associated proteins, including ALP, COL-1, OPN, and OC, is also significantly inhibited [43]. As shown in the results of the present study, although the addition of Wnt-3a under HG conditions markedly enhanced the osteogenic differentiation capacity of BMSCs compared to the HG group, it still failed to fully restore their osteogenic potential to the level observed in the NG group. Furthermore, research has revealed that initiating the Wnt signaling pathway relies on its interaction with the LRP5/6 receptor, which plays a crucial role in mediating downstream signal transduction. DKK1 is recognized as a secreted glycoprotein that interacts with the Wnt receptor LRP5/6 on the cell membrane and its coreceptor KREMEN1, forming a trimeric complex. This complex induces rapid receptor endocytosis, reducing LRP5/6 at the cell surface and thereby blocking Wnt signal transmission, effectively antagonizing the Wnt/β-catenin pathway [44]. In this experiment, the β-catenin inhibition group with added DKK1 showed reduced ALP activity compared to the NG group, along with lower levels of osteogenic genes and proteins. As a result of these findings, we have confirmed that the Wnt/β-catenin pathway contributes positively to the osteogenic differentiation of BMSCs.

Although ROS induce varying degrees of impairment to bone metabolism primarily by interfering with the canonical Wnt/β-catenin signaling pathway, β-catenin itself can, in turn, regulate intracellular ROS levels and exert antioxidative effects [45]. This bidirectional interaction suggests that oxidative stress alone may not irreversibly disrupt bone metabolism solely through inhibition of the canonical Wnt pathway. Importantly, the noncanonical Wnt/Ca^2+^ signaling pathway also plays a critical, synergistic role in the suppression of osteogenesis under oxidative stress conditions, further contributing to bone metabolic dysregulation. Studies indicate that HG levels increase intracellular cytoplasmic free calcium ion concentration [Ca^2+^] across various cell types [46, 47]. When intracellular [Ca^2+^] is elevated, it binds to calmodulin (CaM) to form Ca^2+^/CaM, which initiates the activation of CaMK IIs [48]. Although CaMK II serves as a key signaling factor in noncanonical signaling pathways, it is also closely related to the canonical Wnt signaling pathway. Research indicates that in the canonical Wnt pathway, β-catenin complexes with TCF/LEF transcription factors to activate specific osteogenic target gene expression [38]. However, the TAK1-NLK MAPK cascade is activated by CaMK II, where activated NLK phosphorylates serine 421 on HDAC1, promoting HDAC1 and LEF1 binding and inhibiting the β-catenin and LEF1 interaction [49]. NLK also reduces the binding of TCF to β-catenin, inhibiting the association of the β-catenin–TCF/LEF complex with DNA and consequently hindering β-catenin–TCF/LEF-mediated transcriptional activation [50, 51]. In the HG group of this study, increased CaMK II expression aligns with prior findings. KN-93 is one of the most widely used pharmacological inhibitors for investigating the cellular and in vivo functions of CaMK II. It functions by binding directly to the Ca^2+^/CaM complex rather than to CaMK II itself. This interaction prevents the Ca^2+^/CaM complex from associating with CaMK II, thereby effectively inhibiting its activation and downstream signaling [52]. After the addition of the CaMK II inhibitor to the HG group, the osteogenic differentiation capacity of BMSCs was significantly improved. However, it remained inferior to that observed in the NG group. This result may be attributed to the fact that although KN-93 effectively inhibited CaMK II signaling in the noncanonical Wnt pathway, it did not eliminate the detrimental effects of oxidative stress on the canonical Wnt/β-catenin signaling pathway. Consequently, the osteogenic potential of BMSCs could not be fully restored under HG conditions. Further assays on osteogenic outcomes revealed that upon addition of the CaMK II activator CaCl_2_, osteogenic differentiation of BMSCs was inhibited, and transcription of Runx2 and COL-1 mediated by canonical Wnt/β-catenin signaling axis was reduced. These results are associated with the effects of CaMK II on the canonical Wnt/β-catenin signaling pathway as described in the literature. However, the specific mechanisms require further investigation.

To summarize, this study demonstrates that the osteogenic differentiation of BMSCs on titanium surfaces is inhibited under HG conditions. This process of inhibition is mediated by an overproduction of ROS, which markedly downregulates β-catenin expression within the canonical Wnt signaling pathway. Concurrently, ROS appear to promote the activation of CaMK II expression in the noncanonical Wnt pathway. This suggests that ROS exert a dual regulatory effect on these distinct osteogenesis-related signaling cascades. However, treatment with the ROS inhibitor, Mito TEMPOL, significantly restores β-catenin expression and suppresses CaMK II expression, thereby alleviating impaired osteogenic differentiation. In order to gain deeper insights into the molecular mechanisms through which the Wnt/CaMK II/β-catenin pathway influences osteogenic differentiation in BMSCs, activators and inhibitors of Wnt/β-catenin and Wnt/CaMK II were applied separately. It is observed that the Wnt/CaMK II pathway inhibits osteogenesis in BMSCs, whereas the Wnt/β-catenin pathway promotes osteogenesis in BMSCs.

## 5. Conclusions

In summary, HG–induced oxidative stress activates the Wnt/CaMK II pathway while inhibiting the Wnt/β-catenin pathway, which affects BMSC osteogenesis on titanium surfaces. This mechanism could further compromise the osseointegration of titanium implants. These findings offer novel insights into how DM affects implant bone integration, presenting potential treatment targets that may improve implant integration in individuals with diabetes. To improve osseointegration in diabetic patients, the development of innovative implant materials, as well as the exploration of medications that suppress excessive ROS production and mitigate their harmful impact on the bone-implant junction are highly promising strategies. Moreover, strategies that upregulate Wnt/β-catenin signaling while suppressing the Wnt/CaMK II pathway may further improve implant osseointegration, thereby overcoming the unique challenges posed by oxidative stress in diabetes.

## Figures and Tables

**Table 1:** List of primer sequences used for qRT-PCR.

Gene	Primer sequence (5′–3′)
*β-Catenin*	Forward: GAGTGCTAGGTGCTGTCT
	Reverse: GAGTTAGGTGCTGTCT

*CaMK II*	Forward: TATCCGCATCACTCAGTACCTG
	Reverse: GAAGTGGACGATCTGCCATTT

*COL-1*	Forward: TCGTGCCTAGCAACATGCC
	Reverse: TTTGTCAGAATACTGAGCAGCAA

*Runx2*	Forward: GACTGTGGTTACCGTCATGGC
	Reverse: ACTTGGTTTTTCATAACAGCGGA

*OPN*	Forward: ATCTCACCATTCGGATGAGTCT
	Reverse: TGTAGGGACGATTGGAGTGAAA

## Data Availability

The data that support the findings of this study are available from the corresponding author upon reasonable request.
